# Predictive Validity of the Total Health Index for All-Cause Mortality Assessed in the Komo-Ise Cohort

**DOI:** 10.2188/jea.18.68

**Published:** 2008-04-10

**Authors:** Hiroaki Asano, Kazuo Takeuchi, Yosiaki Sasazawa, Tetsuya Otani, Hiroshi Koyama, Shosuke Suzuki

**Affiliations:** 1 School of Nursing, Kyoto Prefectural University of Medicine.; 2 Faculty of Education, Saitama University.; 3 Facility for Education, University of Ryukyus.; 4 Department of Public Health, Gunma University Graduate School of Medicine.; 5 NPO International Eco-Health Research Group and Gunma Occupational Health Promotion Center.

**Keywords:** Komo-Ise Cohort, Mortality, Risk, Perceived Health, Prospective Studies, Total Health Index (THI)

## Abstract

**Background:**

The Total Health Index (THI), a self-administered questionnaire developed in Japan, is used for symptom assessment and stress management of employees and others; however, it has not been reported whether it can predict mortality risk.

**Methods:**

The THI, with 12 primary and 5 secondary scales, was applied to a cohort consisting of middle-aged residents in Japan. This study, called the Komo-Ise cohort study, was started in 1993. The scale scores were related to 481 deaths from all causes among 10,816 residents over 93 months. The statistics were tested by the Cox hazard model and adjusted for three background variables (sex, age, and district where the subject resided).

**Results:**

Five of the scales [depression and aggression (primary scales), and psychosomatics, neurotics, and schizophrenics (secondary scales)] indicated significant hazard ratios for mortality. The lowest quintile group of the aggression scale score had the largest hazard ratio of 2.58, compared with the middle quintile group (95% confidence interval: 1.88-3.52). The psychosomatics, neurotic scales and depression scales also had a minimum hazard ratio in the middle quintile group. One of the secondary scales, T1, which represents a somatoform disorder, had a significant linear relationship with the mortality risk, although its proportionality with the cumulative mortality rates was not satisfactory.

**Conclusions:**

Five scales of the THI were significantly related to mortality risk in the Komo-Ise cohort, which could be used for score evaluation and in the personal health advice system of the THI.

## INTRODUCTION

The Total Health Index (THI) is a revised version of the Todai Health Index,^1^^-^^3^ a well-known health questionnaire in Japan. It has 17 scales (12 primary scales and 5 secondary ones) and includes a variety of personal information on health; in addition, it offers advice on the applicant’s physical and mental health status. Its validity and reliability have been confirmed by many studies.^4^^-^^11^ It was used in a number of surveys^12^^-^^21^ and produced valuable results. Further revisions of the THI since 2002 have involved its presentation design, reference population, addition of two more scales, and advisory system.

Well-known health questionnaires have been used in studies to find whether perceived or self-rated health can predict the all-cause mortality risk of a lay population. Examples include: the Medical Outcomes Study 36-Item Short-Form Health Survey (SF-36),^22^ the General Health Questionnaire (GHQ),^23^^-^^25^ the Center for Epidemiologic Studies-Depression Scale (CES-D),^26^^-^^29^ the Geriatric Depression Scale (GDS),^30^ the Self-Rating Depression Scale (SDS),^31^ the Minnesota Multiphasic Personality Inventory (MMPI),^32^ and the Cornell Medical Index (CMI);^33^ but few studies have been conducted using the THI. An epidemiologic project called the Komo-Ise Study^34^^-^^38^ was begun in 1993 in Gunma Prefecture, Japan, in which the cohort was surveyed using the THI and other instruments. All deaths were investigated until 2000.

In the Komo-Ise cohort study, the following relationships have been reported: between perceived health and all-cause mortality,^34^ social networks and all-cause or cause-specific mortality,^35^^,^^36^ body mass index and all-cause mortality,^37^ and the two newly developed secondary scales of the THI and all-cause mortality.^38^ The relationships between the scales of THI and mortality risk had been investigated. It will be reported in the present paper, for example, to discover whether or not the top quintile of depressed persons has the highest mortality risk. This is the first report focusing on the predictive validity of the THI for all-cause mortality.

## METHODS

### Subjects

The subjects were from the cohort of the Komo-Ise Study. In brief, the cohort is made up of registered residents aged 40-69 years from Komochi Village and the two downtown districts in Isesaki City, approximately 100 km north of Tokyo, Japan. According to the 1995 census, the populations of the village and the city numbered 12,141 (population density: 292/km^2^) and 120,236 (1,815/km^2^), respectively. The sampled residents of the village and the downtown districts, who were 40-49 years old, numbered 4,875 and 7,755, respectively. A set of self-administered questionnaires that included the THI was distributed to all of the sample residents in the two areas by their respective municipal government offices (in January 1993 for the village and in October 1993 for the city). A total of 4,501 residents in Komochi Village and 7,064 residents from Isesaki City responded (response rate, 92.3% and 91.1%, respectively). A total of 11,565 residents (5,630 men and 5,935 women; response rate, 91.6%) constituted the baseline cohort. The questionnaire items included the respondent’s name, sex, address, date of birth, household size, body height and weight, blood pressure, work hours per week, hours of sleep, and the 130 items from the THI questionnaire. The study was approved by the institutional review board of ethics of the Gunma University Graduate School of Medicine, Maebashi, Japan.

### The Total Health Index (THI)

The Todai Health Index that was developed in 1974^1^^-^^3^ consists of 130 questions on lifestyle, personal preferences, physical symptoms, and mental-condition-related complaints. For example, the question “Do you have headaches?” may be answered by choosing one of the three prepared responses: (1) often; (2) sometimes; or (3) hardly ever or never, to which a scale score of 3, 2, or 1 point(s) is assigned, respectively. Completed THI questionnaires were factor-analyzed repeatedly and 11 factors were extracted, to which a lie scale was added. The factors formed 12 primary scales as follows: many subjective symptoms (SUSY), respiratory symptoms (RESP), eye and skin symptoms (EYSK), mouth and anal symptoms (MOUT), digestive organ symptoms (DIGE), impulsiveness or short temper (IMPU), a lie or social desirability scale (LISC), mental instability (MENT), depression (DEPR), aggression (AGGR), nervousness (NERV), and irregularity of life (LIFE). Each scale includes 10 questions (with a few exceptions) and the score from each question is summed to form a scale score, which ranges from 10 to 30 points. A person’s scale score can be evaluated in a cumulative percentile distribution of a scale score from the reference population.

In addition, the THI differentiates among three types of patient groups [i.e., psychosomatics (PSD), neurotics (NEURO), and schizophrenics (SCHIZO)] and three discriminant function values have been developed^39^^,^^40^ as a linear combination of the primary scales. In 2002, processing software called “THI Plus” was developed,^41^^-^^43^ two secondary scales were added, another reference population was added, and the name was changed from the Todai Health Index to the Total Health Index (THI), although the system of scale scoring and the discriminant functions remained unchanged. The two additional scales were integrated scales^38^^,^^43^ named T1 and T2, which were developed by applying principal component analysis^44^ to the 12 primary scales. T1 was the first component and T2 the second one. The higher the T1 score, the more symptoms and psychological distress there are, suggesting a somatoform disorder,^45^ and vice-versa. A higher T2 score indicates many physical problems without mental distress; and a lower T2 score indicates more serious mental distress without physical problems. The practical validity and reliability of the integrated scales have been studied previously.^38^^,^^46^^,^^47^

### Follow-up

The number of deaths, migrations, and censored cases from the cohort were the results of the survey between 1993 and 2000 obtained from the Basic Residents Registers of each municipality.

### Data for Analysis

Respondents were excluded if three or more questions in any one scale were unanswered, so all of the scales analyzed had none or less than two missing responses each. As a result, 327 participants (7.3%) from the village and 422 (6.0%) of the participants from the city were excluded, the total number of subjects analyzed being 10,816 (93.5%) of the cohort. The scale scores were calculated by giving two points to each one or two missing responses for each completed THI questionnaire. As the score for each response is 1, 2, or 3 and the number of missing responses in a THI questionnaire was two or less, the difference in the scale scores between a fully-completed questionnaire and one that had missing responses was two points or less.

The authors recorded 481 deaths and 368 leaving the area in the data from 1993 through 2000, on which the analyses were conducted on each subject on a month-by-month basis. The starting point was set on January 1, 1993 for the village residents and on October 1, 1993 for the city dwellers, while the date of death or migration from the area was recorded towards the end of the survey period (October 31, 2000 for village residents and August 31, 2000 for city residents).

### Statistical Analyses

The Cox proportional hazards model^48^^,^^49^ was used to assess the relationship between the scale scores and the death rate. Adjustment variables used were not only for sex and age but also for district of residence (city or village), because the city cohort was more likely to engage in evening activities as part of their lifestyle and migrated more than the villagers. As moderate correlations (|r|=0.3 to 0.7, r being the Pearson’s correlation coefficient) were observed among the 12 primary scales and as the secondary scales (PSD, NEURO, SCHIZO, T1, and T2) were obtained as a linear combination of the primary scales, we hesitated to include more than two scales at the same time in the model because of co-linearity problems. Moreover, because there was a possibility that a curve relationship between the THI scales and mortality might occur and log-linearity, which is an important assumption of the model, might not be satisfied, we used a set of dummy variables (D1 to D5) in place of a THI scale. The dummy variables were obtained from the five quintiles of the scale: D1=1 if the scale score was between the 0th and 19th percentile and D1=0 otherwise, and so on (D2, D3, D4, and D5 corresponded to 20-39, 40-59, 60-79, and 80-100 percentile classes, respectively). Using only one set of dummy variables adjusted for the three background factors in every set, 17 analyses were conducted. As the dummy variables were not linearly independent, we used four of them for the calculations and obtained the beta-coefficients for all, with one for the remaining variable being zero. We finally adjusted them so that the minimum value would be zero by adding a constant to all of them. If the maximum value of the coefficients was statistically significant and the proportionality of the cumulative death rate was confirmed, the maximum hazard ratio (MHR) and the 95% confidential interval (CI) were calculated to assess the effect of the scale, in terms of MHR=exp (the maximum coefficient). The proportionality was ascertained by checking whether or not the changes in odds ratios of the cumulative death rate per observation period by year between the highest and lowest risk groups were nearly constant. Statistical analyses were performed with the statistical package SPSS^®^ 12.0j for Windows. When *P* was less than 0.05, the value was considered to be statistically significant.

## RESULTS

Table 1 shows the cases of death or migration by sex, district, and age (classified by each decade in age). Mortality was higher in males than in females and increased with age. The difference in mortality between the city and the village was small. The migration rates were higher for the city than the village.

**Table 1.** All-cause mortality and out-migration rate of the Komo-Ise cohort^*^ by sex, age and district.

District^*^	Age (years)	Population	Death	Out-Migration
	
			n (%)	n (%)
Male				
Village	40-49	920	18 (2.0)	30 (3.3)
	50-59	667	33 (4.9)	15 (2.2)
	60-69	552	74 (13.4)	10 (1.8)
	Total	2,139	125 (5.8)	55 (2.6)

City	40-49	957	18 (1.9)	70 (7.3)
	50-59	1,074	57 (5.3)	40 (3.7)
	60-69	1,115	128 (11.5)	18 (1.6)
	Total	3,146	203 (6.5)	128 (4.1)

Female				
Village	40-49	827	14 (1.7)	29 (3.5)
	50-59	678	13 (1.9)	14 (2.1)
	60-69	530	22 (4.2)	10 (1.9)
	Total	2,035	49 (2.4)	53 (2.6)

City	40-49	1,030	6 (0.6)	56 (5.4)
	50-59	1,242	32 (2.6)	41 (3.3)
	60-69	1,224	66 (5.4)	35 (2.9)
	Total	3,496	104 (3.0)	132 (3.8)

*: Middle aged residents population of Village=Komochi Village (rural area) and City=two downtown districts of Isesaki City being one of old cities in Gunma prefecture, Japan, from 1993 through 2000.

Table 2 shows the results of the Cox hazard model with the three background factors: sex, district, and age. The HRs of the males compared with the females and that of the city compared with the village are shown. The effect of aging by 1 year on the beta-coefficient was calculated and shown as the HR of 10 years of aging that was obtained by multiplying the coefficient by 10. The HRs for sex and age were significant (*P*<0.01) but that for the district they were not. Although the effect of district was not significant (*P*-value was approximately 0.05), we included it in the next step of the analyses as a background factor, because the rates of migration were different between the village and the city.

**Table 2.** Effect of background factors, such as sex, age and district, on the all-cause mortality risk of the Komo-Ise cohort* by the Cox hazard model.

Background factor	Hazard ratio (HR)		95% confidence interval		*P*
Sex	M/F ^†^	2.37	1.95	2.87	0.000
Age	+10 years ^‡^	2.46	2.17	2.78	0.000
District	C/V ^§^	1.22	0.99	1.49	0.056

* : The data were obtained from middle-aged residents in Komochi Village and two downtown districts of Isesaki City in Gunma prefecture, Japan from 1993 through 2000.

† : HR of Males (M) compared with Females (F)

‡ : HR of 10 years of aging obtained by using a beta-coefficient per year being multiplied by 10

§ : HR of the Isesaki City compared with Komochi Village

The results of 17 analyses using dummy variables and adjusted for the 3 background factors are shown in Table 3, with the *P*-value for the maximum value of the coefficients. The effects of the background factors in each analysis were almost the same as those shown in Table 2; for example, the HRs for sex (M/F), age (+10 years), and district (C/V) were 2.45, 2.41, and 1.22, respectively, in the model for the aggression scale; therefore these effects were omitted in Table 3. As shown in the same table, 11 of the 17 scales were significant (i.e., *P*<0.05 for the maximum value of the coefficients). In the significant case the cumulative death rate per observation period by year was calculated for the quintile group for which that coefficient was the lowest (upper line) or highest (lower line), as shown in Table 4. The odds ratios of the cumulative death rates between the highest and the lowest quintile groups varied almost continuously with the observation period for five scales -- DEPR, AGGR, PSD, NEURO, and SCHIZO -- but decreased with the period for the other six scales -- SUSY, RESP, MOUT, LIFE, T1, and T2. For the scales in which proportionality was confirmed, we calculated the MHR and the 95% CI (Table 3).

**Table 3.** Effect of the scales of Total Health Index on the all-cause mortality risk by the Cox hazard model adjusted by sex, age and district of the Komo-Ise cohort ^†^ applied by Total Health Index.

Scale ^††^	Coefficient of Dummy Variable ^‡^						Maximum Hazard Ratio (MHR) ^§^		
	
	D1	D2	D3	D4	D5	*P* ^‡‡^	MHR	95%CI	
Primary									
SUSY	0.044	0	0.214	0.239	0.506	**			
RESP	0.023	0	0.264	0.343	0.420	**			
EYSK	0	0.094	0.078	0.278	0.197	ns			
MOUT	0	0.122	0.193	0.184	0.302	*			
DIGE	0.067	0.082	0.260	0	0.159	ns			
IMPU	0.094	0.090	0.176	0	0.244	ns			
LISC	0.269	0.082	0.186	0	0.259	ns			
MENT	0	0.009	0.106	0.061	0.180	ns			
DEPR	0.004	0.050	0	0.122	0.496	**	1.64	1.26	2.14
AGGR	0.947	0.328	0	0.289	0.207	**	2.58	1.88	3.52
NERV	0.159	0	0.261	0.187	0.292	ns			
LIFE	0.043	0	0.187	0.023	0.445	**			

Secondary									
PSD	0.114	0.071	0	0.350	0.564	**	1.76	1.32	2.34
NEURO	0.075	0.149	0	0.289	0.517	**	1.68	1.25	2.24
SCHIZO	0	0.285	0.214	0.102	0.305	*	1.36	1.01	1.81
T1	0	0.147	0.195	0.279	0.507	**			
T2	0.108	0.062	0.220	0	0.396	**			

† : The data were obtained from middle-aged residents in Komochi Village and two downtown districts of Isesaki City in Gunma prefecture, Japan from 1993 through 2000.

‡ : Dummy variables were constructed as to represent each quintile group of scale score in percentile (D1 corresponding to the first quintile group, D2 to the second, and so on), and coefficients were adjusted so that the lowest was zero.

§ : The MHR was calculated as exp (the maximum coefficient) if the *P*-value was significant and the proportionality was confirmed as in Table 4.

†† : Perceived health indices of the THI including SUSY: many subjective symptoms, RESP: respiratory symptoms, EYSK: eye and skin symptoms, MOUT: mouth and anal symptoms, DIGE: digestive organ symptoms, IMPU: irritability, LISC: lie scale or social desirability scale, MENT: mental instability, DEPR: depression, AGGR: aggression, NERV: nervousness, LIFE: irregularity of life, PSD: psychosomatics, NEURO: neurotics, SCHIZO: schizophrenics, T1: the first integrated scale, T2: the second integrated scale.

‡‡ : Compared between the lowest and the highest coefficient class (^*^: *P*<0.05, ^**^: *P*<0.01, ns: not significant)

Analysis was done individually for each index adjusted by three background factors (sex, age and district) of which effects were omitted because they were almost the same as those shown in Table 2.

**Table 4.** Cumulative death rate (%) of all causes of the quintile group with the lowest (upper line) or the highest (lower line) mortality risk per observation period by year of the Komo-Ise cohort^*^.

Scale ^†^	Quintil group	Observation period (year)					

		1	2	3	4	5	6
SUSY	2	0.23	0.70	1.18	2.01	2.48	3.66
	5	1.68	2.60	2.95	3.52	4.41	5.06

RESP	2	0.68	1.27	1.86	2.51	2.96	4.01
	5	1.52	2.28	2.91	3.54	4.28	5.36

MOUT	1	0.48	1.45	1.99	3.02	3.72	4.49
	5	1.26	1.82	2.25	2.75	3.50	4.28

DEPR	3	0.75	1.34	1.78	2.06	2.82	4.12
	5	1.41	2.12	2.79	3.65	4.56	5.62

AGGR	3	0.60	0.98	1.25	1.80	2.52	2.99
	1	2.28	3.49	4.26	5.22	6.25	7.66

LIFE	2	0.62	1.48	2.11	2.93	3.65	4.75
	5	1.32	1.95	2.30	2.70	3.58	4.29

PSD	3	0.70	1.17	1.65	2.03	2.70	3.11
	5	1.58	2.82	3.57	4.17	5.23	6.32

NEURO	3	0.79	1.24	1.64	2.09	2.65	3.50
	5	1.34	2.29	2.92	3.75	4.57	5.66

SCHIZO	1	0.67	1.35	1.71	2.12	2.63	3.27
	5	1.40	2.11	2.96	3.87	4.44	5.84

T1	1	0.42	1.13	1.55	2.36	3.17	4.19
	5	1.49	2.29	2.91	3.48	4.20	5.04

T2	4	0.65	1.16	1.63	2.29	3.14	4.10
	5	1.63	2.56	3.08	3.56	4.37	5.28

* : The data were obtained from middle-aged residents in Komochi Village and two downtown districts of Isesaki City in Gunma prefecture, Japan from 1993 through 2000.

† : Perceived health indices of the THI including SUSY: many subjective symptoms, RESP: respiratory symptoms, MOUT: mouth and anal symptoms, DEPR: depression, AGGR: aggression, LIFE: irregularity of life, PSD: psychosomatics, NEURO: neurotics, SCHIZO: schizophrenics, T1: the first integrated scale, T2: the second integrated scale, and the scales being not significant in Table 3 were omitted.

The coefficients of D1 to D5, corresponding to SUSY, RESP, MOUT, and T1, showed a nearly linear tendency; but those of AGGR, PSD, NEURO, and DEPR suggested a nonlinear relationship and those of LIFE and T2, which varied irregularly, showed alternate up-and-down shifts.

## DISCUSSION

The variables, sex and age, had a considerable effect on mortality rates, although the district showed only a slight effect in the Cox models. Indeed, the results shown in Table 3 changed greatly if sex or age was excluded from the models but little change was noted when the district was excluded. Thus the difference in the D1 to D5 coefficients was less than 0.02 between the models in which the district was included or excluded.

The proportionality of the scale that had the significant maximum coefficient was checked by using the odds ratios of the cumulative death rate per observation period by year (Table 4). Subsequently 5 scales were accepted, in which AGGR had the strongest effect and only this index had a MHR of more than 2.0. The influence of the AGGR scale (MHR=2.58) was at almost the same level as that of sex (HR=2.37) or being older by 10 years (HR=2.46). The HRs of AGGR increased considerably as the scores from the middle percentile group (D3) went up or down showing a reversed character J, which suggests that AGGR had the lowest point of HR or mortality in the middle percentile group (D3). The PSD scale and the NEURO scale had the second and third largest MHRs of 1.76 and 1.68, respectively, showing the character J. In addition, the DEPR scale showed a non-linear relationship with a reversed character L. These results suggest that one must take account of the various types of curved relationships when analyzing those between perceived health indices and mortality risk.

In contrast, the coefficients of the first integrated scale, T1, which may represent the comprehensive perceived health status of a subject, were considered to be linear, because the coefficients of D1 to D5 increased almost linearly as the quintile class increased. However, as the proportionality was not certain from the fact that its odds ratio of cumulative death rates decreased with the observation period by year (3.55, 2.03, 1.88, 1.47, and 1.20), the MHR was omitted from Table 3. The reasons for omitting the MHRs of the other scales -- SUSY, RESP, MOUT, LIFE, and T2 -- were almost the same as those for T1. The proportionality that was not accepted in the present study might be acceptable if the observation period could be divided into shorter terms, if the classification method could be changed (e.g., classifying by tertiles or deciles instead of quintiles], or if the cause of death could be specified.

To confirm the predictive validity of the THI scales, the significant results of this study were compared with those of other studies on the relationship to all-cause mortality for a middle-aged general or lay population. There are few questionnaires that have a scale corresponding to AGGR of the THI and equally few studies have reported on an association between mortality and the scale score in a general population.

The Physical Component Summary score (PCS) and Mental Component Summary score (MCS) of the SF-36 have been associated with significantly higher mortality (HR 1.60 for PCS, *P*<0.001; HR 1.16 for MCS, *P*=0.036; if the score decreased by 10 points).^22^ Psychological distress in the GHQ has been associated with mortality (HR 1.68, *P*<0.05, compared with the positive and negative groups divided by a threshold of 3/4 of the score,^23^ and HR 1.71, *P*<0.05, compared with the top and bottom tertile groups of the score^24^). Psychiatric symptoms in GHQ have been associated with mortality (HR 1.64 for men and 1.58 for women, *P*<0.01, compared with the high and low score groups divided by the conventional threshold of 4/5^25^). The same results were obtained in the present study: the PSD and NEURO scales were significantly associated with mortality (HR 1.76 for PSD and 1.68 for NEURO, if compared with the middle [D3] and the top quintile groups [D5], and HR 1.57 for PSD and 1.56 for NEURO, if compared with the bottom [D1] and the top quintile groups [D5]).

Many studies have shown that persons identified as depressive, not patients, have a significantly higher mortality risk. The CES-D showed that each 1-standard unit increase of the score predicted a 21% increase in the risk of mortality (HR 1.21, *P*=0.001),^26^ or that the highest depressive symptom quintile group had a significantly higher mortality risk when compared with the lowest one (HR 1.15, *P*<0.01).^27^ The GDS showed that women with 6 or more depressive symptoms had a 2-fold increased risk of death (HR 2.14, *P*<0.01) compared with those who had 5 or fewer depressive symptoms.^30^ The Glostrup depression score showed that a 2-SD difference in the depression score was associated with a relative risk of 1.59 (*P*<0.001).^50^ This result was also observed in the present study: the HRs of the top quintile group (D5) for the THI depression score ranged from 1.56 to 1.64 compared with the bottom (D1) to the middle quintile groups (D3). The MMPI showed that those pessimistic individuals who scored in the upper tertile of the distribution had decreased rates of longevity (HR 1.42, *P*<0.05), compared with optimistic individuals who scored in the bottom tertile of the distribution.^32^ On the other hand, Fredman^28^ showed a non-significant HR of 1.77 (*P*>0.05), in comparing women with the highest (25-58) and lowest (0-1) scores of CES-D. Zhang,^29^ comparing the high (>16) and low (<16) score groups of CES-D, showed a significant HR of 1.54 (*P*<0.05) in a diabetic population but a non-significant HR of 1.03 (*P*>0.05) in a nondiabetic cohort. Vogt,^51^ by using an original scale, showed that the HR for the least depressed -- compared with the most depressed tertiles -- was significant for men (HR 0.71, *P*<0.05) but not significant for all subjects (HR 0.91, *P*=0.47).

The relative risk of 2.06 (*P*<0.001) for schizophrenic patients in a homogeneous population in rural Ireland reported by Morgan^52^ corresponded to the MHR of 1.36 for the SCHIZO in the present study, although the high-risk group of our study was the highest quintile group, not of the patients, but of a lay population.

Assessment using the CMI has shown that symptom- and complaint-rich individuals are associated with a higher mortality (HR 1.24, *P*=0.0002, if 15 positive responses were added),^33^ which might be correlated with the significance of the dummy variables corresponding to the T1 and SUSY scales of the THI, although the proportionality of the two scales was not confirmed. For other scales, including RESP, MOUT, LIFE, and T2, of which the coefficient was significant but for which proportionality was not satisfactory, more analyses or studies are necessary to clarify their relationship with mortality. For the EYSK, DIGE, IMPU, LISC, MENT, and NERV scales, the coefficients of which were not significant, the present results were considered to be reasonable when referred to their content.

The beta-coefficients of the dummy variables (D1 to D5) of T1 and T2 were almost the same as those shown in a previous study^38^ in which the subjects were slightly different from those of the present study; thus the Cox proportional hazards model was not checked for the proportionality. The authors applied Cox models to each sex; but the results were unstable because the beta-coefficients varied greatly if several deaths were excluded from the analysis due to a low number of deaths.

There are various ways of using health questionnaires including the THI. One will be an advising system using THI. Seven classes of advice have been prepared for each scale according to the scale score percentile in our system. Delivered and printed advice by the THI-plus software^41^^-^^43^ developed by some of the authors, should make a person’s lifestyle a healthier one. For example, if an applicant is assessed and informed that he is in the highest risk group on a THI scale, it can be a strong motivation for him to change his lifestyle or attitude so that he can move to a healthier group.

The present study revealed that the five scales of the THI were significantly correlated to mortality, which was the lowest in the middle quintile group of scale scores of AGGR, PSD, NEURO, and DEPR. This may be useful information for those who conduct a study based on the questionnaire. The *P*-value of dummy variables of the T1 scale was significant, though its proportionality in the Cox model was not confirmed. The T1 scale would provide valuable integrated information on one’s health, because it was obtained by using the statistical method of principal component analysis, of which the main role is to integrate correlated variables, with an eigenvalue^44^ more than 5 (i.e., it possesses more than five times the information when compared to one of the primary scales). A higher T1 scale score means more subjective symptoms and complaints or a positive sign in perceived health,^38^ which could lead to a higher mortality risk. Exercise or other stress management practices could decrease a T1 score.^34^^,^^35^^,^^36^ This evidence will also be useful if one wants to improve his life style.

## References

[1] Aoki S, Suzuki S, Yanai H A new trial of making a health and personality inventory, THPI. Kodo Keiryogaku 1974; 2: 41-53. (in Japanese) 10.2333/jbhmk.2.41

[2] Suzuki S, Yanai H, Aoki S Development of a new questionnaire, THI. Igaku no Ayumi 1976; 99: 217-25. (in Japanese)

[3] Aoki S, Suzuki S, Yanai H. Development of a new health questionnaire, the Todai Health Index, as a tool for quantitative evaluation of perceived physical and mental health. In: Suzuki S and Roberts RE, eds. Methods and Applications in the Mental Health Surveys: the Todai Health Index. The University of Tokyo Press, Tokyo, 1991: 59-87.

[4] Suzuki S, Aoki S, Kusakari J Relationship between the scales of Cornell Medical Index and the Todai Health Index, Nippon Koshu Eisei Zasshi 1979; 26: 161-8. (in Japanese)

[5] Asano H Fundamental study on health index: Mainly about Todai Health Index (THI). Kyoto Furitsu Ikadaigaku Zasshi 1988; 97: 433-42. (in Japanese)

[6] Suzuki S, Aoki S, Yanai H. The THI Handbook: Methods and Applications of the Todai Health Index. Shinohara Pub. Co., Tokyo, 1989. (in Japanese)

[7] Sato Y, Aoki S, Suzuki S, Higashidani K Changes of subjective complaints by age: a cross-sectional survey of adult female population by the Todai Health Index questionnaire. Minzoku Eisei 1990; 56: 26-46. (in Japanese) 10.3861/jshhe.56.26

[8] Kishida K, Saito M, Aoki S, Suzuki S. Application of the Todai Health Index and Cumulative Fatigue Index for health care in industry. In: Suzuki S and Roberts RE, eds. Methods and Applications in the Mental Health Surveys: the Todai Health Index. The University of Tokyo Press, Tokyo, 1991: 185-95.

[9] Suzuki S, Roberts RE, eds. Methods and Application in Mental Health Surveys: The Todai Health Index. The University of Tokyo Press, Tokyo, 1991.

[10] Kawada T, Kubota F, Ohnishi N, Satoh K. Validity of screening test for the evaluation of depressive state. Sangyo Igaku 1992; 34: 576-7. (in Japanese) 10.1539/joh1959.34.576 1460789

[11] Kawada T, Suzuki S, Kubota F, Ohnishi N, Satou K Content and cross validity of the Todai Health Index depression scale in relation to the Center for Epidemiologic Studies Depression Scale and the Zung Self-Rating Depression Scale. J Occup Health 1999; 41: 154-9. (in Japanese) 10.1539/joh.41.154

[12] Iwao S, Kondo H, Suzuki S Social and cultural adaptation of the Japanese businessmen and their wives in the United States. Nippon Koshu Eisei Zasshi 1981; 28: 69-80. (in Japanese)

[13] Kishida K, Saito M, Hasegawa T, Aoki S, Suzuki S. Application of health questionnaires for health management in small-medium sized enterprises. Sangyo Igaku, 1986; 28: 3-16. (in Japanese) 10.1539/joh1959.28.3 3712831

[14] Saito M, Kishida K, Aoki S, Suzuki S. The relationship between eye strain and health level in automated industrial work as evaluated by the Todai Health Index questionnaire. In: Suzuki S and Roberts RE, eds. Methods and Applications in the Mental Health Surveys: the Todai Health Index. The University of Tokyo Press, Tokyo, 1991: 197-210.

[15] Takeuchi K, Roberts RE, Suzuki S. Depressive symptoms among Japanese and American adolescents. Psychiatry Res 1994; 53: 259-74. 10.1016/0165-1781(94)90054-X 7870847

[16] Kawada T, Shinmyo R, Suzuki S. Effects of regular health practices on subjective evaluation of health. Sangyo Igaku 1995; 36: 57-63. (in Japanese) 10.1539/joh1959.36.2_57 8007436

[17] Kawada T, Suzuki S. The relationship between being satisfied with one’s health, good health practices and personal symptoms of ill health. Sangyo Eiseigaku Zasshi 1995; 37: 161-3. (in Japanese) 10.1539/sangyoeisei.37.3_161 7796307

[18] Hiramatsu K, Yamamoto T, Taira K, Ito A, Nakasone J A survey on health effects due to aircraft noise on residents living around Kadena Airbase in the Ryukyus. J Sound Vib 1997; 205: 451-60. 10.1006/jsvi.1997.1011

[19] Ohta A, Takeuchi K, Yoshiki S, Suzuki S Differences in lifestyle and perceived health in different occupations in a community. J Occup Health 1998; 40: 325-33. 10.1539/joh.40.325

[20] Tajima K, Takeuchi K, Suzuki S Risk factors for liver dysfunction in middle aged men based on four-year health examination data. J Occup Health 1998; 40: 339-44. 10.1539/joh.40.339

[21] Yosiaki S, Takeuchi K, Ohta A, Tajima K, Suzuki S. Relationship between regular exercise and lifestyle, social network, education and subjective symptom in Japanese middle and elderly residents, Nippon Koshu Eisei Zasshi 1999; 46: 624-37. (in Japanese) 10496031

[22] Tsai SY, Chi LY, Lee CH, Chou P. Health-related quality of life as a predictor of mortality among community-dwelling older persons. Eur J Epidemiol 2007; 22: 19-26. 10.1007/s10654-006-9092-z 17216549

[23] Rasul F, Stansfeld SA, Hart CL, Gillis CR, Smith GD. Psychological distress, physical illness and mortality risk. J Psychosom Res 2004; 57: 231-6. 10.1016/S0022-3999(03)00618-4 15507248

[24] Robinson KL, McBeth J, Macfarlane GJ. Psychological distress and premature mortality in the general population: a prospective study. Ann Epidemiol 2004; 14: 467-72. 10.1016/j.annepidem.2003.11.007 15301783

[25] Huppert FA, Whittington JE. Symptoms of psychological distress predict 7-year mortality. Psychol Med 1995; 25: 1073-86. 10.1017/S0033291700037569 8588004

[26] Everson-Rose SA, House JS, Mero RP. Depressive symptoms and mortality risk in a national sample: confounding effects of health status. Psychosom Med 2004; 66: 823-30. 10.1097/01.psy.0000145903.75432.1f 15564345

[27] Gump BB, Matthews KA, Eberly LE, Chang YF; MRFIT Research Group. Depressive symptoms and mortality in men: results from the Multiple Risk Factor Intervention Trial. Stroke 2005; 36: 98-102. 10.1161/01.STR.0000149626.50127.d0 15569872

[28] Fredman L, Magaziner J, Hebel JR, Hawkes W, Zimmerman SI. Depressive symptoms and 6-year mortality among elderly community-dwelling women. Epidemiology 1999; 10: 54-9. 10.1097/00001648-199901000-00010 9888280

[29] Zhang X, Norris SL, Gregg EW, Cheng YJ, Beckles G, Kahn HS. Depressive symptoms and mortality among persons with and without diabetes. Am J Epidemiol 2005; 161: 652-60. 10.1093/aje/kwi089 15781954

[30] Whooley MA, Browner WS. Association between depressive symptoms and mortality in older women. Study of Osteoporotic Fractures Research Group. Arch Intern Med 1998; 158: 2129-35. 10.1001/archinte.158.19.2129 9801180

[31] Takeida K, Nishi M, Miyake H. Mental depression and death in elderly persons. J Epidemiol 1997; 7: 210-3. 10.2188/jea.7.210 9465545

[32] Brummett BH, Helms MJ, Dahlstrom WG, Siegler IC. Prediction of all-cause mortality by the Minnesota Multiphasic Personality Inventory Optimism-Pessimism Scale scores: study of a college sample during a 40-year follow-up period. Mayo Clin Proc 2006; 81: 1541-4. 10.4065/81.12.1541 17165632

[33] Logue EE, Aubert RE, Knowles MG, Tyroler HA. Symptom reports and mortality in an occupational cohort. J Occup Med 1986; 28: 849-54. 10.1097/00043764-198609000-00016 3772563

[34] Ohta A, Aoki S, Takeuchi K, Yosiaki S, Suzuki S. Lifestyle and sociodemographic risk factors for death among middle-aged and elderly residents in Japan from a five-year follow-up cohort study. J Epidemiol 2001; 11: 5l-60. 10.2188/jea.11.51 11388493 PMC11638024

[35] Iwasaki M, Otani T, Ohta A, Yosiaki S, Kuroiwa M, Suzuki S. Rural-urban differences in sociodemographic, social network and lifestyle factors related to mortality of middle-aged Japanese men from the Komo-Ise cohort study. J Epidemiol 2002; 12: 93-104. 10.2188/jea.12.93 12033534 PMC10468338

[36] Iwasaki M, Otani T, Sunaga R, Miyazaki H, Xiao L, Wang N, . Social networks and mortality based on the Komo-Ise cohort study in Japan. Int J Epidemiol 2002; 3: 1208-18. 10.1093/ije/31.6.1208 12540724

[37] Hayashi R, Iwasaki M, Otani T, Wang N, Miyazaki H, Yosiaki S, . Body mass index and mortality in a middle-aged Japanese cohort. J Epidemiol 2005; 15: 70-7. 10.2188/jea.15.70 15930802 PMC7851061

[38] Asano H, Takeuchi K, Sasazawa Y, Otani T, Koyama H, Suzuki S Characteristic and availability of the integrated scales of Total Health Index (THI). Kosei No Shihyo 2007; 54(5): 1-8.

[39] Aoki S, Suzuki S, Yanai H Identification of psychosomatics, neurotics and schizophrenics by a paper health questionnaire, The Todai Health Index (THI). Igaku no Ayumi 1979; 110: 763-8. (in Japanese)

[40] Suzuki S, Kawa M, Aoki S, Yanai H, Saito Y, Hosoki T Identification of neurotics and schizophrenics from the respective reference normal group by a paper health questionnaire, the Todai Health Index. Kodo Keiryogaku 1979; 6: 28-38. (in Japanese) 10.2333/jbhmk.6.2_28

[41] Asano H Introduction of a computer software for health surveys, Kyoto Furitsu Ikadaigaku Kangogakka Kiyo 2002; 11: 163-8. (in Japanese)

[42] Suzuki S. Outline of the 3rd Edition of the THI_Plus System for Health Check. Takeda Press, Fujisawa, 2005. (in Japanese)

[43] Suzuki S, Asano H, Aoki S, Kurihara H. THI_Plus System for Health Check: Fundamental Data for Usage and Evaluation. Takeda Press, Fujisawa, 2005. (in Japanese)

[44] Okuno T, Haga T, Kume H, Yoshizawa T. Methods of Multi-variate Analysis. Nikkagiren Press, Tokyo, 1971. (in Japanese)

[45] American Psychiatric Association. DSM-IVR, Diagnostic and Statistical Manual for Mental Disorders, 4th revised edition. APA, Washington DC, 1995.

[46] Asano H, Takeuchi K, Sasazawa Y, Koyama H, Suzuki S Distributions of indices of Total Health Index (THI) on the new standard population. Kosei No Shihyo, 2005; 52(7): 1-7. (in Japanese)

[47] Suzuki S ed. A compilation of cases by the Total Health Index. Research Report of Gunma Occupational Health Promotion Center, Maebashi, 2006. (in Japanese)

[48] Cox DR Regression models and life tables. J R Stat Soc B 1972; 34: 187-220.

[49] Maetani S. Clinical Survival Analysis. Nankodo Press, Tokyo, 1996. (in Japanese)

[50] Barefoot JC, Schroll M. Symptoms of depression, acute myocardial infarction, and total mortality in a community sample. Circulation 1996; 93: 1976-80. 10.1161/01.CIR.93.11.1976 8640971

[51] Vogt T, Pope C, Mullooly J, Hollis J. Mental health status as a predictor of morbidity and mortality: a 15-year follow-up of members of a health maintenance organization. Am J Public Health 1994; 84: 227-31. 10.2105/AJPH.84.2.227 8296945 PMC1615016

[52] Morgan MG, Scully PJ, Youssef HA, Kinsella A, Owens JM, Waddington JL. Prospective analysis of premature mortality in schizophrenia in relation to health service engagement: a 7.5-year study within an epidemiologically complete, homogeneous population in rural Ireland. Psychiatry Res 2003; 117: 127-35. 10.1016/S0165-1781(03)00002-7 12606015

